# Patients' Experiences with Specialist Care via Video Consultation in Primary Healthcare in Rural Areas

**DOI:** 10.1155/2014/143824

**Published:** 2014-08-24

**Authors:** Annette M. Johansson, Inger Lindberg, Siv Söderberg

**Affiliations:** Division of Nursing, Department of Health Science, Luleå University of Technology, 971 87 Luleå, Sweden

## Abstract

*Introduction*. Video consultation (VC) can improve access to specialist care, especially for individuals who live in rural areas that are long distances from specialist clinics. *Aim*. The aim of this study was to describe patients' experiences with specialist care via VC encounters. *Method*. Interviews were conducted with 26 patients who had participated in a VC encounter. The data were analysed using thematic content analysis. *Result*. The analysis resulted in two themes. The theme “confident with the technology” was constructed from the categories “possibilities and obstacles in using VC encounters” and “advantages and disadvantages of the technology.” The theme “personal satisfaction with the VC encounters” was constructed from the categories “support from the healthcare personnel,” “perceived security,” and “satisfaction with the specialist consultation.” *Conclusion*. The patients who did not think that the VC was the best care still considered that the visit was adequate because they did not have to travel. An important finding was that the patients' perceived even short distances to specialty care as expensive journeys because many patients had low incomes. Among the patients who had more than one VC, the second encounter was perceived as safer. Additionally, good communication was essential for the patient's perception of security during the VC encounter.

## 1. Introduction

Video consultation (VC) can improve access to specialist care, especially for individuals who live in rural areas [1, 2]. Video consultation is a specialised type of telemedicine that uses technology to provide real-time visual and audio patient assessment at a distance [3]. This system permits a direct interaction between the patient and healthcare personnel because they are separated by space, not time [4]. In this method of meeting, the specialist physician changes the location of care by connecting patients and specialist physicians virtually instead of through the conventional face-to-face contact. Video consultation improves the quality of care and benefits the healthcare organisation [1]. The virtual meeting can provide opportunities to gain access to relevant expertise, without the significance of geographic distance. Video consultation offers opportunities to disseminate and receive knowledge so that misunderstandings can be avoided and/or solved immediately [5]. It is also an opportunity to enhance the local service provision, provides more rapid specialist assessment, and reinforces general practitioners' (GPs) clinical assessments and increases access to and support from the specialist physician [6]. In VC encounters between diabetes patients and endocrinologists, Fatehi et al. [2] found that the specialist physicians mostly were confident with their own recommendations. A lack of physical exam was not considered a limiting factor, and if necessary, the GP could perform the physical examination.

Traveling to healthcare centres is often difficult for individuals with poor health, disabilities, and/or low income. This difficulty can be amplified if the individual has chronic problems that require regular visits to specialist care. If the individual has a disability, the individual may need an escort and/or special transportation, which increases the cost for both the individual and the healthcare system. Visiting a healthcare provider for children is much more expensive because of the need for an accompanying individual [7]. Providing services directly to patients who live in the areas beyond the usual service boundaries are beneficial for hospitals and saves money because of the reduced travel costs [6].

Video consultation in real time allows the simultaneous and rapid exchange of information, which is advantageous if the conversations are complex or of an urgent nature, whether it is a patient consultation or a case discussion [7]. Because of the need for visual assessment in skin examinations, VC can be a valuable tool in the diagnosis and management of dermatologic diseases, especially in rural areas located long distances from specialist care [4]. An accurate and comprehensive medical history can easily be communicated via VC, but if there are circumstances that require a physical examination for a successful assessment, VC may be less suited. Therefore, collaboration and confidence in the assessment by an individual qualified to perform and report the findings are most likely important components for VC success [3].

Agha et al. 2009 [8] suggested that the use of the latest technology can be translated into “quality care” for some patients. Additionally, the attention from two healthcare professionals may promote a patient's confidence [9]. To build trust and patient satisfaction with care, it is important that the patient has confidence in the healthcare provider's clinical skills [8]. There is a continuing need for larger studies of telemedicine that use controlled interventions and telemedicine innovations that involve complex processes and an ongoing collaboration. As the field is rapidly evolving, new knowledge is constantly needed. Understanding the patients' experiences from a qualitative perspective is an area of great interest [10]. Therefore, this study focused on the patients' experiences with specialist care via VC.

### 1.1. Context

This study was performed in northern Sweden. The county of Norrbotten is the largest county in Sweden; it covers one-quarter of the country's total land area but has fewer than 250,000 inhabitants, that is, 2.6% of the Swedish population. Most inhabitants live within a few miles of the largest city in the county, and the central hospital is situated close to the largest city; the rest of the county is sparsely populated [11]. This study area was chosen because the County Council of Norrbotten was planning to implement the use of VC encounters for specialist care in all health centres in the county. Five primary healthcare (PHC) centres reported that they were interested in participating in the study. The patient came to their PHC centre and together with the GP they had encounter with the specialist physician via video consultation.

The distance to the nearest hospital was between 23 kilometres and 220 kilometres (*m* = 119 kilometres).  Table 1 shows the estimated cost/savings (based on ten VC) for the patient, the County Council [12], and also the environment [13]. It is not taken into account that in the winter there can be many delays due to snow and unploughed roads and this means that it is not certain that the patient will make it to be the specialist appointment in time.

## 2. Aim of the Study

The aim of this study was to describe patients' experiences with specialist care via VC encounters.

## 3. Methods

### 3.1. Design

A qualitative design and thematic content analysis were chosen to achieve the aim of the study.

### 3.2. The Intervention: Video Consultation

Healthcare personnel at each PHC centre were given information about the study. They were also given education on the equipment and how to perform video consultation. The technology consisted of a desktop speaker, a microphone, a Logitech camera, and Polycom CMA desktop videoconferencing software. A pilot test was performed in order to train the healthcare personnel to become secure in how the technology worked.

### 3.3. Participants and Procedure

Twenty-six patients were interviewed, including 11 men and 15 women between 18 and 83 years of age (Md = 63, *m* = 59) (Table 2).

The following inclusion criteria for the participants were used: individuals who had participated in VC encounters at the PHC centre with a specialist (dermatologist *n* = 1 or specialist nurse *n* = 1 in heart disease) at the main hospital. The GP provided an information letter about the study and a letter of informed consent to the patient after the VC. The first author contacted the patients who agreed to participate by phone and established a time and place for the interview. Before the interview, the patient received verbal information about the study and the voluntary participation. The interviews were conducted at the PHC centre, in the patient's home or via phone.

### 3.4. Data Collection

Individual semistructured interviews were conducted with the patients. An interview guide was used and contained the following questions: “please tell me about your experience with the VC,” “tell me what you think VC might mean for your future care,” “please share your thoughts on specialist care via VC,” “please tell me what opportunities and obstacles you can see with VC,” “can you tell me which areas of care you think VC at a distance would be helpful for you,” “please tell me how was your experienced about the support from the personnel and why it was good or bad,” “what did you feel about the answers/advice you got from the specialist physician,” “would you consider having VC alone in the room if the staff were available outside if needed,” and “how long did you have to wait before the VC encounter.” Clarifying questions were also used, for example, “can you give some examples,” “how do you think now,” “what do you think it would mean for you,” and “what could have been done differently.” The interviews were performed between June 2010 and December 2011, and they lasted between 10 and 30 minutes (md = 15 minutes), were audio taped, and later were transcribed verbatim.

### 3.5. Data Analysis

A qualitative thematic content analysis was used to analyse the interview texts. According to Baxter [14], thematic analysis is the most complex method because the researcher's interpretations are based on a holistic analysis. Themes are threads of meaning that emerge from the categories. The transcribed interview text was read through several times to obtain a sense of the whole. Then, units of analysis, including words, phrases, sentences, or whole text that corresponded with the aim of the study, were identified and encoded by content, for example, security, advantages, disadvantages, and so forth. The encoded units of analysis were sorted into categories based on the similarities or differences in content. The categories were then sorted into two themes with five categories (Table 3); a theme can be described as threads of meaning that appeared in categories after categories. The researches discussed the categories and themes in order to reach a common outcome. When the categorisation was finished, the units of analysis were reread and checked for the accuracy of their categorisation.

## 4. Results

The analysis revealed two themes with five categories. The themes and categories are presented and illustrated with quotations from the interviews.

### 4.1. Confident with Technology

The theme “confident with the technology” was constructed from the categories “possibilities and obstacles in using VC encounters” and “advantages and disadvantages of the technology.”

#### 4.1.1. Possibilities and Obstacles in Using VC Encounters

Participants described that the VC encounters were a good first step for meeting a specialist physician and receiving a diagnosis was possible from this encounter. Most participants considered there were few or no barriers in using VC, but some participants thought face-to-face encounters would be more appropriate than VC encounters in certain situations. Participants emphasised the importance of the VC equipment being placed in a room where no other individuals, other than the participants in the VC encounter, were present. The obstacles that the participants described were related to the physicians; for example, if the picture quality was poor, the specialist physician's ability to make a diagnosis was affected. Therefore, the participants thought a face-to-face meeting would be better;

*It is not good that the computer is placed in a room where people often enter because someone forgot to press the “busy” key [Participant 15].*

Participants thought that a possible disadvantage of having VC encounters could be that the specialist physician could not experience the patient's problems the same way as they would when meeting face-to-face. Several participants described being disappointed in not having the choice to participate in a VC encounter because of the GP's reluctance to use the technology. The reason for this reluctance was that the GP had no experience with the VC equipment or was not interested. Some participants described that they have not had the opportunity of a VC encounter since it was ambulatory GPs at the PHC. Consequently, the patient booked a new appointment when their regular GP returned to work, so they could have a VC encounter instead of traveling a long distance for a specialist consultation. Another obstacle that the participants highlighted was that VC encounters were not possible at all the PHC centres. Some participants said that they wanted to have the opportunity for a VC encounters in the future, so that they would not have to drive to the hospital by themselves when they are older;

*The thing that was bad was that the ambulatory physician did not want to deal with the video consultation, so we waited for our regular GP [Participant 20].*

#### 4.1.2. Advantages and Disadvantages of the Technology

The majority of participants reported that they saw technology as a major advantage that saved resources;

*I see it (technology) as a big plus and one that saves resources [Participant 25].*

Most participants that used VC encounters considered the picture and sound quality to be good. They were pleased that the specialist physician could see clearly despite the long distance. There were also participants that did not think that the technology worked well. The sound was described as a problem by a few of the elderly participants who had impaired hearing. They said that the participants should be able to hear the specialist physician and suggested that a headset could be provided. In some cases, the GP and the nurse had to explain what was said during the VC encounter because the participant could not hear what was said;

*It had very bad sound, so the nurse and the doctor who was with me explained what she (specialist physician) said; they could provide better sound for older people [Participant 3]. *

Some participants expressed that the VC encounters were slightly abnormal because the picture of the specialist physician was very small and was located at the bottom corner of the computer screen. They said that a larger picture would make it feel more like a face-to-face conversation. There were times when the participants could not see the computer screen because it was turned the wrong way, and they did not understand that they could request that the screen should be repositioned. A short cable to the camera also caused some patients to sit/lie in awkward positions to provide a good image of the problem;

*It was a little awkward to get your feet up on the table because the camera cable was too short, but I managed to do it [Participant 26]. *

Participants described that the nurse took care of the technology, and the GP informed the specialist physician about the participant's problem. This was not considered negative but was merely a statement of fact. Participants who participated in VC encounters described several areas of use for the technology. They considered the VC encounters as an opportunity to contact a specialist physician for a less severe disease or ailment that did not require hospitalisation. Some areas they described included external ailments and injuries, psychiatry, and various medical conditions, such as diabetes, heart disease, and medication adjustments. Participants expressed that the VC encounters should be used based on the patient's problem;

*It depends entirely on what you have as an ailment; certain ailments cannot be dealt with via computer [Participant 5].*

### 4.2. Personal Satisfaction with the VC Encounters

The theme “personal satisfaction with the VC encounters” was constructed from the categories “support from the healthcare personnel,” “perceived security,” and “satisfaction with the specialist's advice.”

#### 4.2.1. Support from the Healthcare Personnel

Participants described that they were well supported by the personnel during the VC encounters. In general, a district nurse and a GP were present with the participant during the VC encounter. The GP was considered to provide support mainly by explaining the problem to the specialist physician, as the GP had access to dates and past medical history that could be difficult for the participant to remember. The support from the district nurse was primarily because she took care of the participant and explained what would happen during the VC encounter. None of the participants had thought about whether VC affected their privacy. VC encounter was seen as an opportunity to receive help with treatment and/or a diagnosis, even if they had to undress for the camera. Participants expressed that they would consider participating alone in the VC encounters with the specialist physician if the GP first responded to the specialist physician's questions and the participant was able to handle the camera as needed;

*I do not see it as a hindrance to have to undress in front of the camera, but I see it as an opportunity to get help [Participant 17].*

Most participants described the VC encounters as different, but these encounters were not viewed negatively. They experienced the VC as a personal encounter, although the meeting occurred via a computer;

*Of course it feels different when you're talking to a computer screen, but it was not negative [Participant 5].*

The VC encounter with the specialist physician was described in positive terms and was perceived as a three-part conversation. Some participants described that the VC encounters were similar to face-to-face meetings. However, some participants also expressed that the VC encounters felt strange or slightly artificial and were not the same as talking to a human. Participants also described that they considered that the specialist physician could see the entire individual in a different way during face-to-face meetings compared with the VC encounters. Although the patients had concerns about the VC encounters, they were considered positive experiences by the participants;

*I thought it was quite personal anyway, even though we were not sitting in the same room but communicated via computer [Participant 7].*

#### 4.2.2. Perceived Security

Participants expressed that they felt secure when they were allowed to choose for themselves whether to participate in the VC encounters or receive a referral;

*I had to choose [to have a VC or not], and it's good that I, as a patient, get to choose [Participant 4].*

Participants who had tried VC encounters previously described that they felt safer the second time, as they had knowledge of the procedure. Participants were pleased to have the GP or specialist's statements repeated or explained immediately what they did not hear or understand. Participants felt secure during the VC encounters if the visit was performed at their own PHC centre in the presence of personnel they recognised and were familiar with. Participants that met the GP for the first time during the VC encounter described that they felt sidelined, as they had not previously met the GP at the PHC centre. However, security decreased if many individuals were present in the room during the VC encounter. Participants felt uncomfortable if their medical problem was located in an intimate place, even if they knew the GP well. In these situations, the participants preferred to be alone during the VC encounter. Participants described that having the opportunity to talk to a specialist physician made them feel content and secure, particularly when it was not possible to meet the same GP, due to a lack of permanent GPs at their PHC centre. Participants felt uncomfortable with the technology if they had to conduct the VC alone in a room. However, they would conduct the meeting alone if the personnel began the VC encounter and was then available outside. However, the participants expressed the importance of having personnel in the room for more severe diseases or if the patient so desired;

*It felt good mentally when you got to talk to the specialist; we do not have our own GP, so we meet the same ambulatory physician just twice [Participant 6].*

#### 4.2.3. Satisfaction with the Specialist Consultation

Most participants were very satisfied with the VC encounters and experienced that they were helped by the specialist physician's advice and diagnosis. They also considered they received answers to their questions, and they believe they would have received the same answer if they had met the specialist physician in person;

*I do not think I would have any other help if I met with the specialist face-to-face [Participant 1].*

Participants who did not receive a diagnosis via the VC were pleased with the tests that were ordered by the specialist physician and that they would later be provided with answers. Participants felt that they would discover and treat their disease more quickly through the VC encounters. Participants experienced that VC encounters at the PHC centre meant increased access to specialist care. Video consultation was considered a smooth, easy, and positive method to receive specialist care. Participants were very satisfied with the short waiting time as VC encounter meant compared to waiting for a referral to a face-to-face meeting. The patients could wait up to several months for a face-to-face visit, whereas they only had to wait a few days before they could participate in the VC encounter with the specialist physician.

If the participant was not helped by the treatment regime suggested during the VC encounter, the GP recommended that the patient go to the specialist physician for a face-to-face visit instead of performing another VC encounter. Participants were worried about not being seen as a person because of the technology. Most participants were very pleased with the VC encounter and wanted to use it as needed for the same or other problems. They felt that, whatever the problem, having the specialist physician “come to the patient” via a VC encounter was better than the patient traveling to the specialist physician;

* It does not matter what condition you have; it has to be smoother with VC than to transport people to the specialist physician [Participant 17].*

The greatest benefit that the participants experienced was that they did not have to travel an entire day to meet with the specialist physician. They said that they were pleased to not have to travel, even if the specialist physician was located nearby. Participants described the VC encounter as a financial gain if travel was considered expensive despite receiving travel grants. Participants expressed satisfaction with not having to take time off from work for a whole day;

*It means a lot for future care to avoid taking time off from work for a whole day [Participant 20].*

They thought VC encounters would be beneficial in their future when they will have difficulty moving because of age and disorders. Video consultation encounters also allowed individuals to save time, and individuals considered that VC benefited the environment. Participants expressed great optimism in not having to need referrals for some specialist areas in the future;

*For my future care, it [VC] means I will get faster help when I do not need a referral [Participant 18].*

They reported that if the diagnosis could not be made at the VC encounter, they considered it to be more quickly to go to a specialist physician for a face-to-face visit. Participants who had met the specialist physician face-to-face considered the follow-up meetings through VC encounters as advantageous. They also considered that VC encounters saved time for the GP.

## 5. Discussion 

Patients described that VC encounter was a good first step for meeting a specialist physician and receiving a diagnosis. This finding is inconsistent with the study by Johansson et al. [15] in which healthcare personnel considered that the first meeting with the specialist physician should be face-to-face. According to Harrison et al. [16], VC encounters can improve patient satisfaction by increasing access to healthcare and can be a tool to increase patient choice about where to have their specialist care. Whitten and Mair [17] described that patients found the new technology exciting. In our study, the majority of participants saw the technology as a major advantage that saved resources, and they considered that the picture and sound quality was adequate. Liu et al. [18] reported that both patients and physicians considered VC encounters as a good idea. In our study the participants with impaired hearing described that if there was problem in hearing, the GP or nurse explained what was said, so that was no problem for them.

In our study, the participants reported that they considered the specialist physician's ability to make a correct diagnosis affected if the picture was not sufficiently clear. In these situations, they considered that a face-to-face meeting would have been better. Gray et al. [7] found that the quality of the video picture and sound was critically important for efficient communication and patient acceptance of VC encounters. Furthermore, the participants described that they were well supported by the GP during the VC encounter. Harrison et al. [16] found that patients liked having their GP present during the VC encounters. Although the GP's role in the VC encounter was minor, the mere presence of the GP gave the patient confidence. In this study, the participants expressed that receiving an immediate explanation of what they did not hear or understand was helpful. According to Hjelm [19], our perception of what we see on the computer screen is influenced by our experience of watching TV; therefore, VC encounters might not be experienced as being real. This perception may cause elderly patients to not accept that the physicians can see and listen to them properly. Participants expressed that they would conduct the VC encounter alone, but because they felt uncertain about the technology, they wished to have healthcare personnel nearby. According to Johansson et al. [15], the healthcare personnel should be comfortable with the technology to ensure patient safety during VC encounters. Additionally, the assessments performed in VC must be clinically reliable and comparable to the usual methods used to deliver care. Incorrect diagnosis can lead to misstatements and possibly fatal consequences [7].

Most participants described the VC encounter as a positive experience and thought of the encounter as a three-part conversation. According to Liu et al. [18], appropriate communication between the physician and patient was important to develop a good relationship and to achieve a positive outcome for the patient. Participants described that the support from the GP during the VC encounter included the GP explaining the patient's medical history and the patient's current problem. Harrison et al. [16] reported that most patients liked that the GP and the specialist physician used medical terminology. They thought that this communication provided a better presentation of their problem compared with what they could have done by themselves. Liu et al. [18] reported that patients did not find any difference in satisfaction between face-to-face encounters and VC encounters. The only difference was the number of requests for repeated utterances; the repetition was much higher for VC encounters compared with face-to-face encounters [18, 20]. This result may have been affected by the patients having VC encounters and face-to-face meetings in the same day to compare VC versus face-to-face encounters.

The results show that the participants were satisfied with the specialist consultation via VC. The VC encounters reduced waiting time, enhanced access to specialist care, and reduced travel. This result concurs with several studies (e.g., [7, 18, 21]) that examined patient satisfaction with VC encounters. Participants described that meeting the specialist physician via VC was better than traveling a long distance. Harrison et al. [16] and Gordon [22] reported that patients appreciated the improved convenience and punctuality associated with VC encounters compared with the outpatient clinics. Mair and Whitten [21] also found that patients who had used VC encounters had the impression that the examinations were more thorough, and the benefits of VC were clear. However, patients experienced an emotional distance between themselves and the specialist. In this study, participants' experiences were consistent with the study performed by Liu et al. [18] in which a VC encounter was less time consuming than a face-to-face consultation. This finding is also consistent with Gordon [22], who found that receiving medical care via VC encounter was easier and that the patients did not think that they would have received better care if they had met the specialist physician face-to-face.

Limitation of the study may be that only two specialist areas were included. But it is questionable whether the outcome would have been different if more areas had been included, since the results were very uniform between the two specialities. The number of participants in this study can be considered as many given that it is a qualitative study and that is a strength of the study. Strength of the study is also that it extends over a long time.

## 6. Conclusion

A majority of the participants considered the VC encounter as a positive experience and thought of the encounter as a three-part conversation. Even for the patients who did not believe the VC encounter provided the best care, the patients still considered VC as a possible option because they did not have to travel. This shows that VC is a possible solution to increase accessibility to specialized care in rural areas. The patients who completed VC encounters more than once perceived the second encounter as safer. This finding may be because these participants had knowledge of how the meeting would proceed. Additionally, good communication was essential for the patient's perception of security during the VC encounter. Another important finding was that even short distances to the specialist physician were perceived as expensive, particularly because many patients had low incomes. Further research should be conducted to investigate whether district nurses responsible for the VC encounter can improve the access to specialist care in rural areas when regular GP is not available. There is also need for studies if VC can increase access to specialist care in other specialities. Also research about the economic and environmental factors need to be examined in larger scale to see the gains VC can provide.

## Figures and Tables

**Table 1:** Overview of savings in time, costs, and environmental impact calculated in 10 VC.

Healthcare centre	A	B	C	D	E	Total
Distance^1^	440 km	46 km	260 km	310 km	136 km	1192 km
Travel time^1^	5 h 30 min	45 min	3 h 30 min	4 h 30 min	2 h	14 h 15 min
Number of visits	10	10	10	10	10	10
Number off km (*n* = 10)	4400 km	460 km	2600 km	3100 km	1360 km	11920 km
Saved journey (*n* = 10) time	55 h	7 h 30 min	35 h	45 h	20 h	162 h 30 min
Travel (*n* = 10) allowance^2^	4400 SEK	4600 SEK	2600 SEK	3100 SEK	1360 SEK	16 060 SEK
Patient petrol expenses/journey^3^ (*n* = 10)	792 SEK (7920 SEK)	83 SEK (828 SEK)	468 SEK (4680 SEK)	558 SEK (5580 SEK)	245 SEK (2448 SEK)	2 146 SEK (21 456 SEK)
Carbon dioxide kg (*n* = 10 journeys)	612 kg^5^	64 kg^5^	362 kg^5^	431 kg^5^	189 kg^5^	1658 kg^5^
	588 kg^6^	61 kg^6^	347 kg^6^	414 kg^6^	182 kg^6^	1592 kg^6^

^1^Back and forth.

^2^County Council provides compensation of 10 SEK/10 km if traveling by own car.

^3^Calculated on patient expenses 18 SEK/10 km (the County Council give 10 SEK in travel allowance).

^4^Gasoline.

^5^Diesel.

**Table 2:** Demographic data.

Gender male (*n* = 11)/age	Gender female (*n* = 15)/age
33–46–59–60–64–70–72–74–78–81–83	18-19–25–39–42–49–59–61–63–68–72-73–77-77–82

**Table 3:** Overview of themes and categories from the interviews (*n* = 27).

Theme	Categories
Confident with technology	Possibilities and obstacles in using VC encounters
	Advantages and disadvantages of the technology

Personal satisfaction with the VC encounters	Support from the healthcare personnel
	Perceived security
	Satisfaction with the specialist consultation
